# The Southern Surgical and Gynecological Association

**Published:** 1900-01

**Authors:** 


### THE SOUTHERN SURGICAL AND GYNECOLOGICAL ASSOCIATION.

THE twelfth annual meeting of the Southern Surgical and Gynecological Association, held in New Orleans December 5th, 6th and 7th, was one of the best meetings in the history of the Association. To say this is equivalent to saying that it was one of the best meetings ever held in this country, for the Southern Surgical and Gynecological Association is regarded at home and abroad as one of the best special organizations in the world, and her volumes of transactions are much sought after, and are inferior to none.

The papers presented, as a rule, were of much scientific and practical value; the discussions, free and untrammeled, of almost equal value.

That the meeting was held in New Orleans need only be stated to let every one know that the Association was royally entertained as only New Orleans can entertain. A luncheon at the Boston Club, an evening at the French Opera, a ride on the Mississippi to one of the large sugar plantations, complimentary tickets to the races, were some of the delightful entertainments to the members of the Association as a body. Numerous private breakfasts, luncheons and dinners were given to selected parties by the resident members. Dr. Lewis, as chairman of the Committee of Arrangements, made every one at home by his cordial hospitality. The local members of the profession were untiring in their efforts for the success of the meeting. A most commendable feature of all these entertainments was that they were so arranged as not to interfere with the published program. Atlanta will find it hard to equal New Orleans. There are no politics in the Southern Surgical and Gynecological Association; one rarely hears the subject of election of officers, or selection of place of meeting mentioned until the nominations are made. The Association owes much of its success to this total absence of medical politics. The officers elected were:

President—Dr. A. M. Cartledge, Louisville, Ky.

Vice-Presidents—Dr. Manning Simons of Charleston, S. C., and Dr. W. P. Nicolson of Atlanta, Ga.

Secretary—Dr W. E. B. Davis of Birmingham, Ala.

Treasurer—Dr. W. D. Haggard, Jr., of Nashville, Tenn.

The selection of Atlanta as the next place of meeting was without solicitation, and met the unanimous approval of the Association. Atlanta is to be congratulated. It is an honor of which any city should be proud to have the privilege of entertaining so distinguished a body of specialists.

The management of the St. Charles Hotel (headquarters of the

Association) contributed in every legitimate way to the success of the meeting and the entertainment of the members. Special rates were made to those attending the meeting. *Especial* attention was shown those to whom *special* rates were made. This is a commendable example, which should be emulated by other hotel proprietors in the different cities where the future meetings will be held.

Dr. Joseph Tabor Johnston of Washington, D. C., presided with unusual grace, dignity and impartiality.

Dr. A. M. Cartledge of Louisville, Ky., the President-elect, commands the personal friendship and professional esteem of every member of the Association on account of his magnetic personality and his professional ability.

Any mention of the Southern Surgical and Gynecological Association would be incomplete without a reference to Dr. W. E. B. Davis of Birmingham, the father of the Association, the man to whose untiring efforts and faithful devotion the continued success of the Association has been largely due. As Secretary, Dr. Davis has borne the burdens of the Association with unflagging zeal and ever increasing efficiency.

Every physician in Georgia who is entitled to membership in this Association should avail himself of the privilege.

---

THE Georgia Physicians owe a vote of thanks to the noble Governor of our State who recently showed pure unadulterated backbone and intellectual equipment sufficient to expose by veto the fallacies of the *Osteopathy Bill.* This is one case where the man was greater than his party. The last Georgia Legislature would afford cartoons enough to last *Puck* and *Judge* a hundred years had the artists only been present. A more blooming set of flagrant idiots could scarely be found. They passed every ridiculous bill that their ingenuity could devise, and passed absolutely nothing that would be valuable. The osteopaths of the West, especially their school in Kirksville, Mo., are sending runners all over the South to lobby with the various State Legislatures to aid them in legalizing a private enterprise. They found a lot of the most gullible suckers in this State Legislature that they could conceive of in their wildest fancy. But thanks it is that we have a *man* at the helm who has sense enough to keep our State from being the laughing-stock of the whole country. Governor Candler is the right man for Governor, and the physicians appreciate his recent effort for the Medical Profession. The following reasons for vetoing the bill, as given by Governor Candler, are unanswerable and show the clear head of our chief executive:

I withhold my approval from this bill because, aside from the objection that it advertises one particular school—the American School of Osteopathy of Kirksville, Mo.—there is no necessity for such an enactment. It provides among other things for the creation of another medical examining board in the State. There are already three of these boards and any graduate of any lawfully chartered medical college may go before either of them, present his diploma and be examined. If he passes an examination satisfactory to the board, a board the members of which are selected because of their eminence in the profession and their skill in medical science, he is authorized to practice medicine anywhere in this State, and apply any treatment he may deem best, including the methods of osteopathy. If he is not a graduate of a reputable medical college and cannot pass a satisfactory examination in the usual branches of medical education, he ought not to be licensed to engage in the practice of medicine.

R.

---

THE Georgia Medical Association meets in Atlanta next April. This is mentioned because we wish to call the attention of the medical profession to the necessity of making preparations to be present. The profession should stand by their State Medical Societies, and endeavor both by their presence and the contribution of papers to make it well thought of and recognized by the laity.

The tone of the medical profession throughout the State is largely dependent upon the influence exerted over it by the State society. We must stand together if success in every respect is to be obtained. Prepare your papers and let our next meeting be worthy of this old State and worthy of our State Society.

---

WE wish to call the attention of our exchanges to the fact that the present name of this Journal is the ATLANTA JOURNAL-RECORD OF MEDICINE, and would request that in sending matter to us the above title be used. The present Journal is a consolidation of the *Atlanta Medical and Surgical Journal* and the *Southern Medical Record,* and yet our exchanges continue to send two sets of Journals. We wish all to take note of this fact.

---

THE following officers have been elected in the Atlanta Society of Medicine for 1900:

President—Dr. J. C. Olmsted.
Vice-President—Dr. T. V. Hubbard.
Secretary—Dr. Claude A. Smith.
Treasurer—Dr. E. Van Goidtsnoven.
Librarian—Dr. M. Hoke.

---

AGNES NACKER is reported by the *Medical News* as the first woman doctor admitted to general practice in Germany. She resides in Berlin. It has taken the Prussian Cabinet two years to decide her case.

